# Posttraumatic Stress Disorder and Obstructive Sleep Apnea in Twins

**DOI:** 10.1001/jamanetworkopen.2024.16352

**Published:** 2024-06-24

**Authors:** Amit J. Shah, Viola Vaccarino, Jack Goldberg, Minxuan Huang, Yi-An Ko, Xin Ma, Oleksiy M. Levantsevych, Nicholas L. Smith, Nikila Alagar, Iman Mousselli, Dayna A. Johnson, Gari D. Clifford, J. Douglas Bremner, Donald L. Bliwise

**Affiliations:** 1 Department of Epidemiology, Rollins School of Public Health, Emory University, Atlanta, Georgia; 2 Department of Medicine, Emory University School of Medicine, Atlanta, Georgia; 3 Atlanta Veterans Affairs Healthcare System, Decatur, Georgia; 4 Seattle Epidemiologic Research and Information Center, Office of Research and Development, Department of Veterans Affairs, Seattle, Washington; 5 Department of Epidemiology, University of Washington, Seattle; 6 Department of Pediatrics, Stanford University, California; 7 Department of Biostatistics and Bioinformatics, Rollins School of Public Health, Emory University, Atlanta, Georgia; 8 Department of Biostatistics, Mailman School of Public Health, Columbia University, New York, New York; 9 Department of Biomedical Informatics, Emory University School of Medicine, Atlanta, Georgia; 10 Wallace H. Coulter Department of Biomedical Engineering, Georgia Institute of Technology and Emory University, Atlanta, Georgia; 11 Department of Psychiatry and Behavioral Sciences, Emory University School of Medicine, Atlanta, Georgia; 12 Department of Radiology and Imaging Sciences, Emory University School of Medicine, Atlanta, Georgia; 13 Department of Neurology, Emory University School of Medicine, Atlanta, Georgia

## Abstract

**Importance:**

Obstructive sleep apnea (OSA) is a common condition in older adult (aged >65 years) populations, but more mechanistic research is needed to individualize treatments. Previous evidence has suggested an association between OSA and posttraumatic stress disorder (PTSD) but is limited by possible selection bias. High-quality research on this association with a careful evaluation of possible confounders may yield important mechanistic insight into both conditions and improve treatment efforts.

**Objective:**

To investigate the association of current PTSD symptoms and PTSD diagnosis with OSA.

**Design, Setting, and Participants:**

This cross-sectional study of twin pairs discordant for PTSD, which allows for adjustment for familial factors, was conducted using in-laboratory polysomnography from March 20, 2017, to June 3, 2019. The study sample comprised male veteran twins recruited from the Vietnam Era Twin Registry. The data analysis was performed between June 11, 2022, and January 30, 2023.

**Exposure:**

Symptoms of PTSD in twins who served in the Vietnam War. Diagnosis of PTSD was a secondary exposure.

**Main Outcomes and Measures:**

Obstructive sleep apnea was assessed using the apnea-hypopnea index (AHI) (≥4% oxygen saturation criterion as measured by events per hour) with overnight polysomnography. Symptoms of PTSD were assessed using the PTSD Checklist (PCL) and structured clinical interview for PTSD diagnosis.

**Results:**

A total of 181 male twins (mean [SD] age, 68.4 [2.0] years) including 66 pairs discordant for PTSD symptoms and 15 pairs discordant for a current PTSD diagnosis were evaluated. In models examining the PCL and OSA within pairs and adjusted for body mass index (BMI) and other sociodemographic, cardiovascular, and psychiatric risk factors (including depression), each 15-point increase in PCL was associated with a 4.6 (95% CI, 0.1-9.1) events-per-hour higher AHI. Current PTSD diagnosis was associated with an adjusted 10.5 (95% CI, 5.7-15.3) events-per-hour higher AHI per sleep-hour. Comparable standardized estimates of the association of PTSD symptoms and BMI with AHI per SD increase (1.9 events per hour; 95% CI, 0.5-3.3 events per hour) were found.

**Conclusions and Relevance:**

This cross-sectional study found an association between PTSD and sleep-disordered breathing. The findings have important public health implications and may also enhance understanding of the many factors that potentially affect OSA pathophysiology.

## Key Points

**Question:**

What is the association of posttraumatic stress disorder (PTSD) with obstructive sleep apnea (OSA) after accounting for acknowledged medical risk factors for OSA?

**Findings:**

In a cross-sectional study of 132 older male veteran twins discordant for PTSD undergoing in-laboratory polysomnography, current PTSD diagnosis and symptoms were associated independently with OSA, even after controlling for demographics, behavioral factors, cardiovascular risk factors, and familial factors.

**Meaning:**

These findings suggest that PTSD may be an independent, novel, and heretofore unrecognized risk factor for OSA in older men.

## Teaser

This cross-sectional study investigates the association of current posttraumatic stress disorder (PTSD) symptoms and PTSD diagnosis with obstructive sleep apnea in older adult male twins.

## Introduction

Obstructive sleep apnea (OSA) is typically considered to be a medical condition with multiple, often overlapping risk factors, including advanced age, obesity, male sex, upper airway narrowing, genetics, and cardiovascular disease.^1^ Despite the wealth of evidence that has uncovered these OSA risk factors, the role of psychiatric disorders that might disrupt sleep, such as posttraumatic stress disorder (PTSD), is debated and has important clinical and public health implications given that both are increasingly prevalent conditions.^2,3^ In addition, both PTSD and OSA are associated with increased cardiovascular disease risk, which underscores the public health importance of this research.^4,5^

Previous studies of the association between PTSD and OSA have yielded inconclusive results primarily due to methodological constraints.^6,7,8,9,10,11,12,13,14^ Many have examined symptomatic patients who were referred for clinical evaluation of possible OSA, which could bias the sample; individuals with and without PTSD could differ in the likelihood of reporting symptoms and seeking care. Furthermore, several investigations relied on PTSD assessments based on medical record review or self-report, which may not be as accurate as a formal clinical assessment. Genetic and familial factors (eg, early life socioeconomic status) may play an important role in each condition and, as such, may lead to attenuated estimates when not fully considered in models.^15,16^ Finally, many studies lacked an adequate control sample without PTSD.^6^

To overcome these limitations, we evaluated the association of PTSD with OSA in a controlled sample of male veteran twins who underwent a formal psychiatric and polysomnography (PSG) evaluation as part of the Emory Twin Study Follow-Up.^17^ This sample was not selected based on referral for either PTSD or sleep disturbance.^18^ We were also able to estimate the influence of PTSD with high internal validity by comparing PTSD-discordant brothers who shared genetic and familial characterstics.^15,16,19^ We tested the hypothesis that veteran twin males with current PTSD symptoms or a clinical diagnosis of PTSD may be more likely to have obstructive apneic episodes and hypoxia compared with their brothers with fewer PTSD symptoms or without a PTSD diagnosis. We also assessed self-reported sleep disturbance to confirm the well-established association between PTSD and poor-quality sleep.

## Methods

### Study Cohort

This cross-sectional study is a substudy based on a follow-up of the Emory Twin Study that was conducted from March 20, 2017, to June 3, 2019.^4,17^ Twin participants at baseline were selected from the Vietnam Era Twin Registry, a large national sample of adult male twins aged 61 to 71 years who served on active duty during the Vietnam War era (1964-1975).^20^ Twin pairs participated together on the same day to minimize measurement error. All twins signed a written informed consent, and the Emory University institutional review board approved the study. We followed the Strengthening the Reporting of Observational Studies in Epidemiology (STROBE) reporting guideline.

### Measurement of PTSD

We obtained a clinical diagnosis of PTSD using the Structured Clinical Interview for DSM (SCID) for the *Diagnostic and Statistical Manual of Mental Disorders* (Fourth Edition).^21^ Following the diagnostic algorithm, PTSD was classified as either current (met criteria in previous month) or remitted (did not meet criteria in previous month). We also examined current PTSD symptom severity using the self-administered PTSD checklist (PCL) for the *Diagnostic and Statistical Manual of Mental Disorders* (Fifth Edition), which has strong internal consistency, test-retest reliability, and convergent and discriminant validity.^22^ A PCL score of greater than 30 (on a scale of 0-80) was found to have a 94% sensitivity and 94% specificity for classifying PTSD diagnosis in a previous validation study.^23^

### Measurement of Sleep Staging and OSA With In-Laboratory PSG

Twins underwent overnight full PSG in the Emory Sleep Center to derive measures of sleep-disordered breathing in a controlled environment. They slept in private rooms and elected bedtimes and wake-up times of their own choosing that were consistent with their home schedule. The day prior to the overnight study, twins were supervised by staff continuously from morning to bedtime. Napping was not permitted. The PSG procedures followed the guidelines of the American Academy of Sleep Medicine.^24^ We recorded respiration with both airflow pressure transducers and thermocouples placed adjacent to the mouth (for oral breathing) and measured finger pulse oximetry throughout the recording. The PSG readings were scored in 30-second epochs by a trained registered polysomnographic technologist masked to all clinical information about the participant. The PSG was performed using the Embla N7000 digital recording system using RemLogic software (Natus). We scored OSA with current American Academy of Sleep Medicine guidelines for apnea (≥90% drop in oronasal sensor signal excursion for ≥10 seconds) and hypopnea (≥30% drop in oronasal sensor signal for ≥10 seconds accompanied by ≥4% drop in oxygen saturation [Sao_2_]).^24^ Apneas and hypopneas were divided by total sleep time to yield the apnea-hypopnea index (AHI), which constituted our primary measure of OSA severity.^25^ The diagnosis of moderate or severe OSA was defined by an AHI of 15 or higher.^26^ The proportion of central apneas (relative to obstructive and mixed apneas) was also tallied for each PSG.

Other indicators of OSA included the respiratory disturbance index, which includes all apneas, hypopneas, and respiratory effort–related arousals per hour of sleep, as well as several measures of hypoxic burden, including the oxygen desaturation index (ODI), defined as the number of drops in Sao_2_ of at least 4% per hour, and the cumulative proportion of sleep spent with Sao_2_<90%, expressed as a percentage of total sleep time. A small number of participants in the sample had been previously diagnosed with OSA and had been prescribed continuous positive airway pressure (CPAP), and a subset of these reported regular use of CPAP in their homes. With approval of their personal physicians, CPAP was not used for the single night of PSG in this study.

### Other Study Assessments

At each visit, in addition to the sleep measures implemented specifically in the current protocol as described earlier, we performed a thorough assessment that included medical history, sociodemographic information, health behaviors, blood pressure, selected blood chemistries, anthropometric measures, and current medications, as previously described.^27^ We collected self-identified race and ethnicity (non-Hispanic Black, non-Hispanic White) via self-report as part of the assessment of the sample demographic. Physical activity was measured using the Baecke Questionnaire of Habitual Physical Activity.^28^ Employment was classified as working full-time or part-time by self-report. History of coronary artery disease that might have occurred from the time of the initial screen was defined as a previous diagnosis of myocardial infarction or coronary revascularization procedures. The SCID administration allowed us to assess lifetime history of major depression and substance abuse in addition to PTSD. Service in Southeast Asia was determined from military records. Zygosity information was assessed by DNA typing as previously described.^29^ Participants completed the Pittsburgh Sleep Quality Index (PSQI), a self-rating scale (0-21, with higher scores meaning worse sleep quality) of general sleep disturbance.^30^

### Statistical Analysis

The data were analyzed between June 11, 2022, and January 30, 2023. Our primary exposure for the study was the PCL score because of the greater number of pairs discordant for PCL symptoms (n = 132, defined by >0 difference in score between brothers) and increased statistical power compared with the 30 twins discordant for PTSD diagnosis. We first compared twin brothers within pairs discordant for PCL symptoms (defined by PCL score difference >0) by calculating prevalence (for binary data only) or mean (SD) values of baseline characteristics in the brother with above (higher PCL twin) vs below (lower PCL twin) the pair mean. These characteristics included sociodemographic factors, health factors, and medications. We then evaluated OSA continuous and categorical measures in both subgroups.

We examined the association between PTSD and OSA within twin pairs discordant for PTSD symptoms or diagnosis, which, by design, controlled for demographic, shared familial, and early environmental influences. This design also reduced the effect of shared exposures during the examination day since twin pairs were examined together.^31^ We used generalized estimating equations, which offer robust methods for SE estimation. In each model, we examined the within-pair differences in sleep outcomes as the dependent variables as a function of the within-pair differences in PTSD symptoms or diagnoses as the independent variables. Within-pair difference terms were calculated by subtracting the twin pair average from the individual value.^32^ We also compared OSA prevalence by current PTSD diagnosis and tested for significant differences using the Fisher exact test. More in-depth information regarding the statistical methods, as well as full outputs of the main models, are provided in the eMethods in Supplement 1.

For our within-pair analyses, we excluded participants who were singletons (the other brother deceased or did not participate) or had concordant or exact same PCL scores (n = 49). Our primary exposure, within-pair PCL difference, was analyzed as a continuous variable. To enhance the applicability of our findings within a clinical setting, we present the estimated OSA outcomes associated with a 15-point within-pair PCL difference (one-half of the 30-point cutoff for PTSD).^23^ Obstructive sleep apnea outcome measures included AHI as the primary outcome and respiratory disturbance index, ODI, and hypoxic burden as secondary outcomes. Our secondary exposure was current PTSD diagnosis based on clinical interview. We examined the prevalence of moderate or severe OSA by PTSD diagnosis group.

We examined 2 multivariable models. The first model included body mass index (BMI, as measured by weight in kilograms divided by height in meters squared) only, given its importance in OSA pathogenesis and relationship with other cardiovascular risk factors. Our full model included traditional cardiovascular disease risk factors as possible mediators in the association between PTSD and OSA; it also included possible confounders such as years of education, employment status, and psychological or behavioral factors, including depressive symptoms, past PTSD, antidepressant use, and alcohol use. The eFigure in Supplement 1 shows a graphical representation of the potential mechanistic and causal pathways involved. We examined both raw PCL score and BMI as well the standardized values to compare their strengths of association with each other in unadjusted and fully adjusted models. For the secondary analysis examining PTSD-discordant pairs only, we did not include all covariates (confounders and mediators) together in a single model to avoid model overfitting due to the limited sample size. Instead, we divided the covariates into 2 separate models. One model included only cardiovascular mediators, while the other included sociodemographic and psychiatric confounders. In addition, we examined for linear dose-response associations of OSA with PCL symptoms among individual twins, including an examination of the association by tertile of individual PCL scores with mean AHI and moderate to severe OSA prevalence. We also evaluated the potential effects of CPAP use and OSA history in additional sensitivity models.

Missing covariate data were rare (<5%); thus, we used all available data without imputation. A 2-sided *P* < .05 was used for statistical significance, and 95% CIs were calculated from model parameters. Statistical analyses were performed using SAS, version 9.4 software (SAS Institute, Inc).

## Results

There were 181 male twins in the total sample (mean [SD] age, 68.4 [2.0] years; 10 Black [6%] and 171 White [94%] race) who underwent overnight sleep testing with PSG, including 74 complete twin pairs. Of these 74 pairs, 66 (42 [63%] monozygotic) were discordant for PCL symptom level, and 15 pairs were discordant for current PTSD. Table 1 compares twins within PCL-discordant pairs (n = 132) separated by whether the twin had the higher or lower PCL score compared with the mean pair score. The mean (SD) PCL score in the lower PCL group was 4.5 (7.0) and 15.4 (14.3) in the higher PCL group. In addition, the SD of the within-pair PCL score difference was 8.1. The brothers who had the higher PCL score showed expected differences compared with the brothers with the lower PCL score, including a higher likelihood of Vietnam combat exposure, smoking or alcohol history, depression, and use of antidepressants. They also had higher PSQI scores and sleep-disordered breathing outcomes.

**Table 1.** Characteristics of 132 Twins Within Pairs, Separated by PCL Score Above (Higher PCL Twin) vs Below (Lower PCL Twin) Pair Average

Characteristic	No. (%)	
	Lower PCL twin	Higher PCL twin
Age, mean (SD), y	68.4 (2.0)	68.4 (2.0)
Male sex	66 (100)	66 (100)
Monozygotic	42 (63)	42 (63)
Race		
Non-Hispanic Black	4 (6)	4 (6)
Non-Hispanic White	62 (94)	62 (94)
Married	48 (73)	47 (71)
Education, y	14.1 (2.6)	13.3 (2.1)
Employed part-time or full-time	15 (23)	16 (24)
Vietnam theater exposure	22 (33)	33 (50)
Body mass index^a^	29.8 (4.4)	29.5 (4.5)
Smoking status		
Ever smoker	40 (61)	46 (7)
Current smoker	12 (18)	10 (15)
History		
Coronary heart disease or angina	4 (6)	9 (14)
Hypertension	37 (56)	39 (59)
Diabetes	11 (17)	10 (15)
Dyslipidemia	43 (65)	45 (68)
Hypertension, mmHg		
Systolic blood pressure	139 (18.8)	141.6 (19.2)
Diastolic blood pressure	79.0 (11.0)	79.1 (12.4)
Lipid profile, mg/dL		
High-density lipoprotein cholesterol	42.9 (12.0)	40.6 (11.8)
Triglycerides	186.4 (76.9)	219 (135.6)
Low-density lipoprotein cholesterol	97.6 (33.3)	97.2 (25.7)
Glucose	109.2 (50.9)	99.1 (14.6)
Medication		
Statin	35 (53)	38 (58)
Aspirin	24 (36)	32 (48)
β-Blocker	13 (20)	16 (24)
ACE-I or ARB	16 (24)	12 (18)
Antidepressant	7 (11)	15 (23)
Baecke activity score (total)	8.0 (1.3)	7.9 (1.3)
Lifetime history of		
Alcohol abuse	13 (20)	2 (3)
Drug abuse	6 (9)	8 (12)
Depression	9 (14)	19 (29)
PSQI total score^b^	5.8 (3.8)	7.1 (3.5)
AHI	16.1 (16.7)	19.5 (20.8)
Respiratory disturbance index^c^	29.9 (21.6)	32.4 (25.1)
Oxygen desaturation index^d^	14.2 (14.7)	18.1 (20.5)
Sleep duration with Sao_2_ <90%, %	6.9 (12.7)	8.5 (14.8)
Obstructive sleep apnea		
Mild (AHI ≥ 5)	48 (73)	50 (76)
Moderate (AHI ≥ 15)	25 (38)	28 (42)
Severe (AHI ≥ 30)	11 (17)	13 (20)
Self-reported history of OSA	11 (17)	14 (21)
Self-reported history of OSA and prescribed CPAP	7 (11)	12 (18)
Central apnea, %	4 (6)	4 (6)

Abbreviations: ACE-I, angiotensin-converting enzyme inhibitor; AHI, apnea-hypopnea index (calculated as number of apneas and hypopneas divided by total sleep time); ARB, angiotensin receptor blocker; CPAP, continuous positive airway pressure; OSA, obstructive sleep apnea; PCL, posttraumatic stress disorder checklist; PSQI, Pittsburgh Sleep Quality Index; Sao_2_, oxygen saturation.

SI conversion factors: To convert high-density and low-density lipoproteins to mmol/L, multiply by 0.0259; to convert triglycerides to mmol/L, multiply by 0.0113; to convert glucose to mmol/L, multiply by 0.0555.

^a^ Calculated as weight in kilograms divided by height in meters squared.

^b^ Scores range from 0-21, with higher scores indicating worse sleep quality.

^c^ Calculated as all apneas, hypopneas, and respiratory effort–related arousals per hour of sleep.

^d^ Calculated as the number of drops in Sao_2_ of at least 4% per hour.

### Sleep-Disordered Breathing

**Figure 1.**
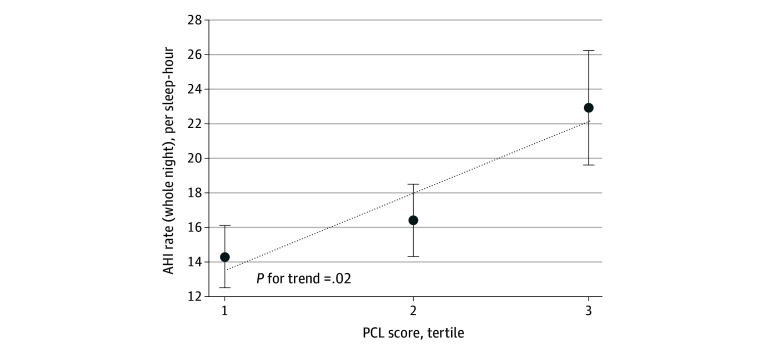
Association of Posttraumatic Stress Disorder Checklist (PCL) Score Tertile Among Individual Twins With Apnea Hypopnea Index (AHI) The PCL tertile was based on PCL scores of less than 2 for the first tertile, 2 to 9 for the second tertile, and greater than 9 for the third tertile (n = 181). The diagonal dotted line indicates the trend slope, and the whiskers indicate the SE.

**Figure 2.**
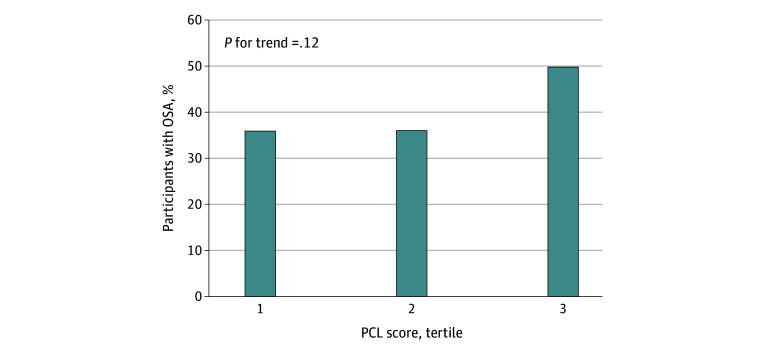
Association of Posttraumatic Stress Disorder Checklist (PCL) Score Tertile Among Individual Twins With Moderate to Severe Obstructive Sleep Apnea (OSA) The PCL tertile was based on PCL scores of less than 2 for the first tertile, 2 to 9 for the second tertile, and greater than 9 for the third tertile (n = 181).

The mean (SD) AHI was 17.7 (14.9) events per hour, and the mean proportion of the night with Sao_2_ less than 90% was 8.9% (16 men). In addition, 74% of the sample (98 men) had at least mild OSA (AHI≥5), 40% of the sample (72 men) had moderate to severe OSA (AHI≥15), and 18% of the sample (33 men) had severe OSA (AHI≥30). Figure 1 shows an increasing mean AHI rate per PCL tertile across twins treated as individuals (tertile 1 [lowest], 14.3 [95% CI, 10.6-18.0]; tertile 2, 16.4 [95% CI, 12.2-20.5]; tertile 3 [highest], 22.9 [95% CI, 16.3-29.4]; *P* for trend = .02), and Figure 2 shows the trends in moderate to severe OSA prevalence by PCL tertile (tertile 1 [lowest], 36%; tertile 2, 36%; tertile 3 [highest], 50%; *P* for trend = .12). Both figures show increased OSA severity and frequency for each incremental step up in PCL tertile, although this trend was significant only for AHI. Within 15 twin pairs discordant for current PTSD, OSA was prevalent in 8 brothers (53%) with current PTSD and 4 brothers (27%) without current PTSD (Fisher exact *P* = .10).

Table 2 and Table 3 present the multivariable analysis for the associations between within-pair differences in PCL scores and current PTSD with OSA outcomes. In fully adjusted models, each 15-point within-pair difference in PCL score was associated with a 4.6 (95% CI, 0.1-9.1) events-per-hour higher AHI, 6.4 (95% CI, 2.1-10.7) events-per-hour higher ODI, and a 4.8% (95% CI, 0.6%-9.0%) greater sleep duration with Sao_2_ less than 90% (Table 2). When examining current PTSD as an exposure variable, the results were consistent with PCL. Most notably, current PTSD associated with an approximately 10-unit higher adjusted higher AHI in separate models involving potential cardiovascular moderators (model 2) (10.5; 95% CI, 5.7-15.3) and sociodemographic and psychiatric confounders (model 3) (10.7; 95% CI, 4.0-17.4) (Table 3). Other OSA-related outcomes showed similar associations with current PTSD, and the PTSD-zygosity interactions were not statistically significant in any of the models. We also evaluated the potential effects of CPAP therapy and OSA history in additional sensitivity models, but no meaningful differences in estimates were found. More detailed information on the results with full model outputs is available in eTables 1 to 5 in Supplement 1.

**Table 2.** Within-Pair Analysis of the Association Between Sleep Outcomes Based on Polysomnography and PCL Score (66 Pairs)

Outcome	Value (95% CI)	
	Model 1^a^^,^^b^	Model 2^a^^,^^c^
AHI, per sleep-h	4.1 (1.0 to 7.1)	4.6 (0.1 to 9.1)
Respiratory disturbance index, per sleep-h^d^	2.5 (−1.6 to 6.6)	1.4 (−5.0 to 7.9)
Oxygen desaturation index, per sleep-h^e^	4.4 (1.5 to 7.3)	6.4 (2.1 to 10.7)
Sleep duration with Sao_2_ <90%, %	4.3 (1.5 to 7.2)	4.8 (0.6 to 9.0)

Abbreviations: AHI, apnea-hypopnea index (calculated as number of apneas and hypopneas divided by total sleep time); PCL, Posttraumatic Stress Disorder Checklist; Sao_2_, oxygen saturation.

^a^ Results are shown as estimates in the regression models per 15-unit increase in PCL score. All models were adjusted for age, sex, and familial factors by design based on modeling within twin pairs discordant for posttraumatic stress disorder symptoms.

^b^ Adjusted for body mass index.

^c^ Adjusted for education, employment, body mass index, history of cardiovascular disease, hypertension, current smoking, diabetes, hyperlipidemia, Baecke physical activity score, Beck Depression Inventory-2 score, remitted posttraumatic stress disorder, history of alcohol abuse, number of alcoholic drinks in the past 30 days, and antidepressant use.

^d^ Calculated as all apneas, hypopneas, and respiratory effort–related arousals per hour of sleep.

^e^ Calculated as the number of drops in Sao_2_ of at least 4% per hour.

**Table 3.** Within-Pair Analysis of the Association Between Sleep Outcomes Based on Polysomnography and Current PTSD Status (15 Pairs)

Outcome	Value (95% CI)		
	Model 1^a^^,^^b^	Model 2^a^^,^^c^	Model 3^a^^,^^d^
AHI, per sleep-h	7.0 (1.7 to 12.4)	10.5 (5.7 to 15.3)	10.7 (4.0 to 17.4)
Respiratory disturbance index, per sleep-h^e^	3.7 (−4.3 to 11.7)	4.0 (−3.0 to 11.1)	10.3 (3.6 to 17.0)
Oxygen desaturation index, per sleep-h^f^	7.6 (1.7 to 13.4)	11.0 (5.3 to 16.6)	12.1 (4.7 to 19.6)
Sleep duration with Sao_2_<90%, %	7.9 (−1.0 to 16.7)	5.8 (−2.2 to 13.8)	16.4 (7.5 to 25.4)

Abbreviations: AHI, apnea-hypopnea index (calculated as number of apneas and hypopneas divided by total sleep time); PTSD, posttraumatic stress disorder; Sao_2_, oxygen saturation.

^a^ Results are shown as estimates comparing current vs never PTSD diagnosis. All models were adjusted for age, sex, and familial factors by design based on modeling within twin pairs discordant for PTSD.

^b^ Adjusted for body mass index.

^c^ Adjusted for history of cardiovascular disease, hypertension, current smoking, diabetes, hyperlipidemia, and Baecke physical activity score.

^d^ Adjusted for education, employment, Beck Depression Inventory-2 score, remitted PTSD, history of alcohol abuse, number of alcoholic drinks in the past 30 days, and antidepressant use.

^e^ Calculated as all apneas, hypopneas, and respiratory effort–related arousals per hour of sleep.

^f^ Calculated as the number of drops in Sao_2_ of at least 4% per hour.

When comparing standardized estimates of PCL and BMI together in mutually adjusted models, we found that each 1-SD increase in PCL score was associated with a 1.9 (95% CI, 0.5-3.3) events-per-hour increase in AHI, while each SD increase in BMI was associated with a 2.6 (95% CI, 1.0-4.2) events-per-hour increase in AHI. However, in fully adjusted models, the estimates changed to 2.1 (95% CI, 0.1-4.2) and 1.7 (95% CI, 0.2-3.2) for PCL and BMI, respectively.

## Discussion

In this cross-sectional study of Vietnam War era veteran twin pairs, we found that within brothers discordant for PTSD symptoms, increased symptoms were associated with statistically and clinically higher AHI. This analysis is, to our knowledge, the most rigorously controlled to date for examining the association between a psychiatric anxiety disorder such as PTSD and OSA, as the twin brothers are matched for demographic, familial, partial genetic, and other early life factors that can otherwise influence both PTSD and OSA.^15^ Studies of discordant twins benefit from high levels of internal validity compared with individual-level analyses, and the focus on within-pair differences may be applicable to clinical settings in which within-person differences in PTSD symptoms before and after treatment are measured.^18,33^ The statistical models were also adjusted for several possible confounders and moderators to estimate the association of PTSD symptoms specifically with OSA severity. The associations were stronger in the fully adjusted models that examined PTSD-discordant pairs (Table 2) than models that examined twins as individuals (Figure 1), which emphasizes the importance of the twin design. With our discordant twin models, we also found that the standardized effect size for PTSD symptoms was remarkably similar to BMI, which is one of the most established risk factors for OSA.^34,35^

Our study is supported by previous work suggesting similar associations, although many previous studies found lower effect sizes that may have been due to methodological limitations.^6^ Most studies have included clinical samples of individuals who were referred for sleep center evaluation because of suspected OSA, and in these studies, individuals with a lower propensity for clinical evaluation could be underrepresented. Such individuals may include, for example, those with mental health conditions, including PTSD, or those with increased barriers to accessing health care, such as financial or transportation.^36^ From this underrepresentation, OSA rates may be underestimated in the PTSD group by restricting the cases to only participants with mild symptoms. Our work also suggests that PTSD may be more strongly associated with OSA than depression, which our group has previously examined with no association being found.^37^ In addition, depression was not associated with OSA in our models (eTables 1-3 in Supplement 1). These negative findings do, however, contrast with previous studies showing positive associations in different populations^38,39^; therefore, more research is needed on the effects of depression and other psychiatric conditions. In light of previous work that included depression, we view our findings as likely to be more specific to the altered breathing pathophysiology accompanying an anxiety disorder such as PTSD rather than accompanying a mood disorder. Despite the modestly small sample size, our use of twin models and the Vietnam Era Twin Registry offers advantages, as the results may be less prone to bias due to clinical symptoms or help-seeking engagement.

Our findings emphasize the need for more studies to examine mechanisms underlying endotypes of OSA that incorporate psychological stress pathways. Possible mechanisms include pharyngeal collapsibility and exaggerated loop gain, which describe the centrally mediated respiratory response to the mild carbon dioxide retention at the onset of sleep.^40^ Posttraumatic stress disorder may cause a lower sleep arousal threshold and decreased autonomic and respiratory reflexes.^40^ Nighttime PTSD symptoms, such as nightmares, may increase sleep fragmentation, which in turn may increase airway collapsibility.^6^ We speculate that specific brain pathways that may be altered in PTSD may also be involved.^41^ This speculation is supported by studies of OSA using functional brain imaging that have shown alterations in regions of the brain involved in stress regulation, such as the thalamus and anterior cingulate cortex.^42^ Neurologic substrates between the brain and the visceral organs that regulate respiration and pharyngeal patency may also be involved.^43^

The clinical relevance of our findings from a psychiatric treatment perspective is supported by studies suggesting that OSA may impair PTSD recovery. For example, previous studies have shown that OSA treatment may help to reduce PTSD symptoms,^44^ although adherence rates are low.^45^ Previous studies have also shown improvement in depressive symptoms with CPAP, further supporting the adjunct benefit that psychiatric interventions may offer.^46^ Symptoms of OSA may also increase anxiety (due to choking sensation) and, therefore, may worsen PTSD symptoms in individuals who already have the condition.^6^ Furthermore, OSA may have global cognitive consequences, which may result in decreased resiliency toward and coping with PTSD symptoms.^9^ As such, OSA may enhance the risk of PTSD in individuals exposed to trauma and may be worsened by low adherence to therapies such as CPAP. Thus, sleep disturbance and PTSD symptoms may accelerate each other in a feed-forward fashion. Our study also suggests a need to examine the effects of PTSD treatment on OSA severity.

### Limitations

Our findings are subject to several limitations. The cross-sectional study design limited our ability to evaluate the directionality of our results and potential causality. Nonetheless, because the PTSD in our sample occurred subsequent to a prior traumatic event (elucidated via SCID), reverse causality (ie, OSA causing PTSD) may be less likely; in other words, OSA may not be a causal factor in incident PTSD within the sample of individuals studied here. Our sample consisted of older, mostly White men; therefore, the results cannot be generalized to other groups. Nonetheless, the high level of consistency has the distinct advantage of increasing the internal validity of the analysis, which is a critical first step for such studies.^18^ Previous research also has suggested that these outcomes are not restricted to men and that they may be similar in women.^47^ The small sample size of participants discordant for current PTSD limited our ability to adjust for all possible confounders in the same models, but the lack of confounding in our larger analysis of PTSD symptoms in which all covariates were included suggests that this association is not otherwise explained by any of the covariates. In addition, the smaller sample size is offset by the strength of using gold standard metrics, such as the structured clinical interview for psychiatric disorders and overnight in-laboratory PSG, which may decrease the risk of misclassification and improve the accuracy of the estimates.

## Conclusions

In this cross-sectional study of veteran twins, we found a strong dose-response association between PTSD and OSA. The twin design allowed close control of familial influences, and the sampling strategy using the Vietnam Era Twins Registry minimized bias. The results suggest that functional neurobiologic and stress pathways modulating respiratory regulation and airway collapse are important in the etiology of OSA. More research is needed to examine possible biobehavioral and psychophysiologic interventions that could ameliorate both sleep-disordered breathing and PTSD.

## Notes

Supplement 1.
eMethods.
**eTable 1.** Association of PCL-5 Score With AHI **eTable 2.** Association of PTSD Status With AHI **eTable 3.** Association of Standardized PCL and BMI With AHI **eTable 4.** Association of PCL-5 Score With AHI, Examining for Interaction With Zygosity **eTable 5.** Association of PTSD With AHI **eFigure.** Directed Acyclic Graph for Multivariable Models

Supplement 2.
Data Sharing Statement
