# Supplementary material for: Posttraumatic Stress Disorder and Obstructive Sleep Apnea in Twins
Source: JAMA Netw Open. 2024 Jun 24;7(6):e2416352. doi: 10.1001/jamanetworkopen.2024.16352 (PMC11197451; doi:10.1001/jamanetworkopen.2024.16352)
Supplement: Supplement 1. — eMethods. eTable 1. Association of PCL-5 Score With AHI eTable 2. Association of PTSD Status With AHI eTable 3. Association of Standardized PCL and BMI With AHI eTable 4. Association of PCL-5 Score With AHI, Examining for Interaction With Zygosity eTable 5. Association of PTSD With AHI eFigure. Directed Acyclic Graph for Multivariable Models [file jamanetwopen-e2416352-s001.pdf]

Supplemental Online Content

Shah AJ, Vaccarino V, Goldberg J, et al. Posttraumatic stress disorder and obstructive sleep apnea in twins. JAMA Netw Open. 2024;7(6):e2416352. doi:10.1001/jamanetworkopen.2024.16352

eMethods

eTable 1. Association of PCL-5 Score With AHI

eTable 2. Association of PTSD Status With AHI

eTable 3. Association of Standardized PCL and BMI With AHI

eTable 4. Association of PCL-5 Score With AHI, Examining for Interaction With Zygosity

eTable 5. Association of PTSD With AHI

eFigure. Directed Acyclic Graph for Multivariable Models

This supplemental material has been provided by the authors to give readers additional information about their work.

© 2024 Shah AJ et al. *JAMA Network Open.*

eMethods

Statistical Methods

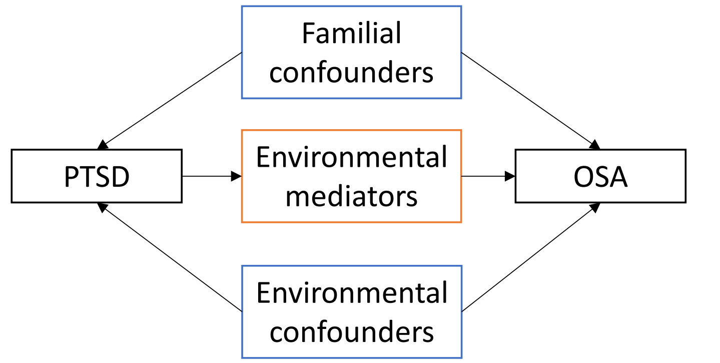

Figure 1: Model of twin models that control for familial confounding by design. Control for environmental factors requires regression models that control for their effects.

We used twin models (figure 1) to help control for familial factors in the investigation of the relationship between PTSD and obstructive sleep apnea. Examples of familial factors include socioeconomic status, family history, parental factors, prenatal and early life experiences, culture, and geography. In addition, because the twins were enrolled together and traveled at the same time, we were able to control for variations due to visit date, staff, season, travel, and other environmental exposures. In monozygotic twin pairs, we can also fully control for genetic factors, while in dizygotic twin pairs, we can only partially control for genetic factors (half, on average). Because of our limited ability to examine differences by zygosity due to the small sample size of discordant twin pairs, we included partial to full genetic effects within the umbrella of familial factors. Not only may unaccounted familial factors partially confound the results, but they may also increase random error that can reduce the precision of the results (reduced internal consistency).

Our analyses focused on complete pairs of twins in which the PCL score differed by at least one point, which we defined as PCL discordant pairs. Therefore, we excluded twin pairs where the scores were exactly the same. In most PCL-concordant cases, both twins had a PCL score of 0.

For each variable used in the analysis, we calculated the pair average, and then the within-pair difference by calculating the difference between the individual twin value and the between-pair mean as shown in figure 2 below. These methods have been described in more detail here: Carlin *et al.*, Int J Epidemiol. 2005 Oct; 34(5):1089-99. Doi: 10.1093/ije/dyi153.

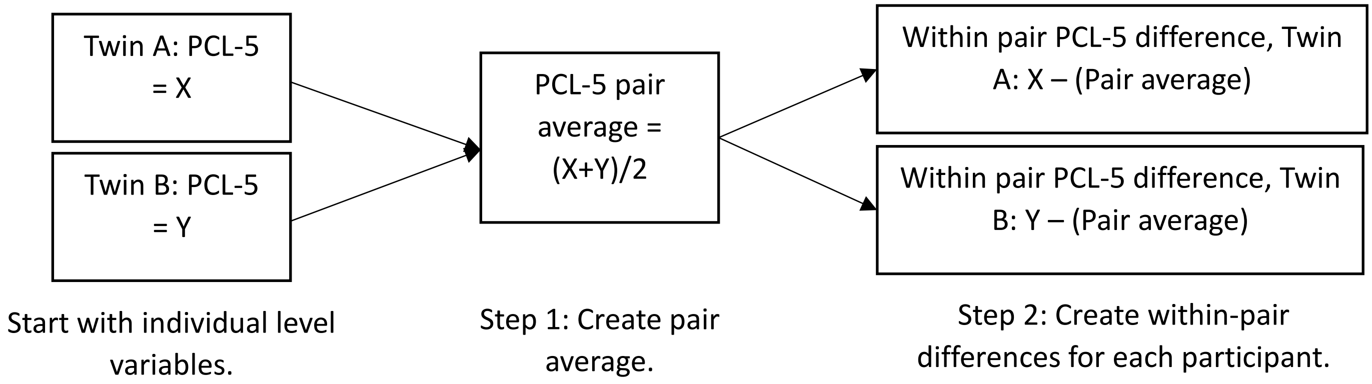

Figure 2 - How we created within-pair difference variables.

© 2024 Shah AJ et al. *JAMA Network Open.*

Similarly, we measured within-pair differences for the co-variates and outcomes. This allowed us to use the following linear regression model using generalized estimating equations, as also discussed by Carlin 2005 (reference above):

Difference in AHI = Intercept + Beta(1)(Within-pair PCL-5 difference) + Beta(2)*(Within pair covariate difference)…*

Our interpretation of this model was that each unit increment of the difference in PCL-5 score (which was re-scaled to 15-point difference to provide a meaningful clinical difference) was associated with a Beta(1) difference in AHI between brothers while controlling by design for demographic, familial and certain environmental confounders, as well as adjusting for covariates in the model.

© 2024 Shah AJ et al. *JAMA Network Open.*

Supplemental Tables

eTable 1. Association of PCL-5 Score With AHI

Table 1a: Association of PCL-5 Score with AHI (fully adjusted, non-standardized)

	Estimate	Lower 95% CI	Upper 95% CI	p
PCL-5 Score, per 15-unit increase	6.4	0.2	12.7	0.04
Body Mass Index (kg/m^2)	1.7	0.2	3.2	0.03
Baecke Physical Activity Scale	-2.0	-3.6	-0.4	0.02
History of CVD	15.9	8.4	23.4	<0.001
Hypertension	10.8	5.5	16.2	<0.001
Current Smoker	-9.1	-16.2	-2.0	0.01
Diabetes Mellitus	0.5	-6.6	7.7	0.88
Hypercholesterolemia	-7.5	-13.2	-1.8	0.01
Remitted PTSD	-0.7	-8.4	6.9	0.85
Years of education	-1.0	-2.1	0.2	0.10
Currently employed	-1.4	-7.1	4.3	0.63
Beck Depression Inventory Score	-0.4	-0.9	0.2	0.19
Alcohol abuse	-0.4	-7.6	6.9	0.92
Antidepressant use	3.3	-4.4	11.1	0.40
Number of alcoholic drinks in the past 30 days	-1.8	-2.7	-0.9	<0.001

© 2024 Shah AJ et al. *JAMA Network Open.*

Table 1b: Association of PCL-5 Score with AHI (fully adjusted, standardized PCL, BMI, and Baecke score)

	Estimate	Lower 95% CI	Upper 95% CI	p
PCL-5 Score (standardized)*	2.1	0.1	4.2	0.04
Body Mass Index (standardized)*	1.7	0.2	3.2	0.03
Baecke Physical Activity Scale	-2.0	-3.6	-0.4	0.02
History of CVD	15.9	8.4	23.4	<0.001
Hypertension	10.8	5.5	16.2	<0.001
Current Smoker	-9.1	-16.2	-2.0	0.01
Diabetes Mellitus	0.5	-6.6	7.7	0.88
Hypercholesterolemia	-7.5	-13.2	-1.8	0.01
Remitted PTSD	-0.7	-8.4	6.9	0.85
Years of education	-1.0	-2.1	0.2	0.10
Currently employed	-1.4	-7.1	4.3	0.63
Beck Depression Inventory Score	-0.4	-0.9	0.2	0.19
Alcohol abuse	-0.4	-7.6	6.9	0.92
Antidepressant use	3.3	-4.4	11.1	0.40
Number of alcoholic drinks in the past 30 days	-1.8	-2.7	-0.9	<0.001

*normalized with mean of zero and each unit being equivalent to one standard deviation

© 2024 Shah AJ et al. *JAMA Network Open.*

eTable 2. Association of PTSD Status With AHI

Table 2a: Association of PTSD Status with AHI, adjusting for psychiatric factors

	Estimate	Lower 95% CI	Upper 95% CI	p
Current PTSD	10.7	4.0	17.4	<0.01
Remitted PTSD	-0.1	-15.3	15.0	0.99
Years of education	1.5	-1.1	4.1	0.26
Currently employed	-11.1	-22.3	0.1	0.05
Beck Depression Inventory Score	0.1	-0.4	0.6	0.61
Alcohol abuse	-11.7	-24.2	0.8	0.07
Antidepressant use	-2.1	-11.5	7.3	0.66
Number of alcoholic drinks in the past 30 days	-0.1	-3.2	2.9	0.93

Table 2b: Association of PTSD Status with AHI, adjusting for cardiac factors

	Estimate	Lower 95% CI	Upper 95% CI	p
Current PTSD	10.5	5.7	15.3	<0.001
History of CVD	16.9	1.2	32.5	0.04
Hypertension	22.9	15.2	30.7	<0.001
Current Smoker	-1.3	-12.7	10.1	0.82
Diabetes Mellitus	-8.8	-18.3	0.7	0.07
Hypercholesterolemia	-15.4	-22.5	-8.3	<0.001
Baecke Physical Activity Scale	1.8	-3.9	7.6	0.53

© 2024 Shah AJ et al. *JAMA Network Open.*

eTable 3. Association of Standardized PCL and BMI With AHI

	Estimate	Lower 95% CI	Upper 95% CI	p
PCL-5 Score (standardized)	2.1	0.1	4.2	0.04
Body Mass Index (standardized)	1.7	0.2	3.2	0.03
Baecke Physical Activity Scale	-2.0	-3.6	-0.4	0.02
History of CVD	15.9	8.4	23.4	<0.001
Hypertension	10.8	5.5	16.2	<0.001
Current Smoker	-9.1	-16.2	-2.0	0.01
Diabetes Mellitus	0.5	-6.6	7.7	0.88
Hypercholesterolemia	-7.5	-13.2	-1.8	0.01
Remitted PTSD	-0.7	-8.4	6.9	0.85
Years of education	-1.0	-2.1	0.2	0.10
Currently employed	-1.4	-7.1	4.3	0.63
Beck Depression Inventory Score	-0.4	-0.9	0.2	0.19
Alcohol abuse	-0.4	-7.6	6.9	0.92
Antidepressant use	3.3	-4.4	11.1	0.40
Number of alcoholic drinks in the past 30 days	-1.8	-2.7	-0.9	<0.001

© 2024 Shah AJ et al. *JAMA Network Open.*

eTable 4. Association of PCL-5 Score With AHI, Examining for Interaction With Zygosity

	Estimate	Lower 95% CI	Upper 95% CI	p
PCL-5 Score	0.6	-1.1	2.4	0.48
Zygosity	-0.2	-3.1	2.8	0.91
PCL-5 Score*Zygosity Interaction	2.0	-0.1	4.0	0.06
Body Mass Index (kg/m^2)	0.8	0.1	1.5	0.02
Baecke Physical Activity Scale	-2.9	-5.3	-0.6	0.01
History of CVD	17.2	9.7	24.7	<0.001
Hypertension	11.6	6.3	17.0	<0.001
Current Smoker	-9.6	-16.6	-2.5	0.01
Diabetes Mellitus	0.9	-6.1	8.0	0.79
Hypercholesterolemia	-8.2	-13.8	-2.5	0.00
Remitted PTSD	0.8	-7.0	8.5	0.85
Years of education	-1.2	-2.3	0.0	0.05
Currently employed	-2.2	-7.9	3.5	0.45
Beck Depression Inventory Score	-0.5	-1.1	0.1	0.09
Alcohol abuse	-0.6	-7.8	6.6	0.87
Antidepressant use	4.5	-3.2	12.3	0.25
Number of alcoholic drinks in the past 30 days	-1.9	-2.8	-1.0	<0.001

© 2024 Shah AJ et al. *JAMA Network Open.*

eTable 5. Association of PTSD With AHI

Table 5a. Association of PTSD with AHI, examining for zygosity interaction and adjusting for cardiac factors

	Estimate	Lower 95% CI	Upper 95% CI	p
Current PTSD	12.9	7.4	18.4	<0.001
Current PTSD*Zygosity Interaction	-8.6	-19.5	2.4	0.13
Zygosity	-0.6	-4.5	3.3	0.76
History of CVD	13.9	-1.6	29.4	0.08
Hypertension	22.0	14.5	29.6	<0.001
Current Smoker	-1.2	-12.1	9.8	0.83
Diabetes Mellitus	-12.7	-23.1	-2.4	0.02
Hypercholesterolemia	-13.5	-20.8	-6.3	<0.001
Baecke Physical Activity Scale	1.4	-4.1	7.0	0.61

Table 5b. Association of PTSD with AHI, examining for zygosity interaction and adjusting for psychiatric factors

	Estimate	Lower 95% CI	Upper 95% CI	p
Current PTSD	10.8	4.1	17.6	<0.001
Current PTSD*Zygosity Interaction	-2.1	-21.4	17.2	0.83
Zygosity	-0.6	-5.9	4.8	0.84
Remitted PTSD	0.1	-15.4	15.5	0.99
Years of education	1.5	-1.2	4.1	0.28
Currently employed	-10.7	-22.4	0.9	0.07
Beck Depression Inventory Score	0.2	-0.5	0.8	0.59
Alcohol abuse	-10.6	-27.5	6.3	0.22
Antidepressant use	-2.1	-11.5	7.3	0.66
Number of alcoholic drinks in the past 30 days	-0.1	-3.2	2.9	0.93

© 2024 Shah AJ et al. *JAMA Network Open.*

eFigure. Directed Acyclic Graph for Multivariable Models*

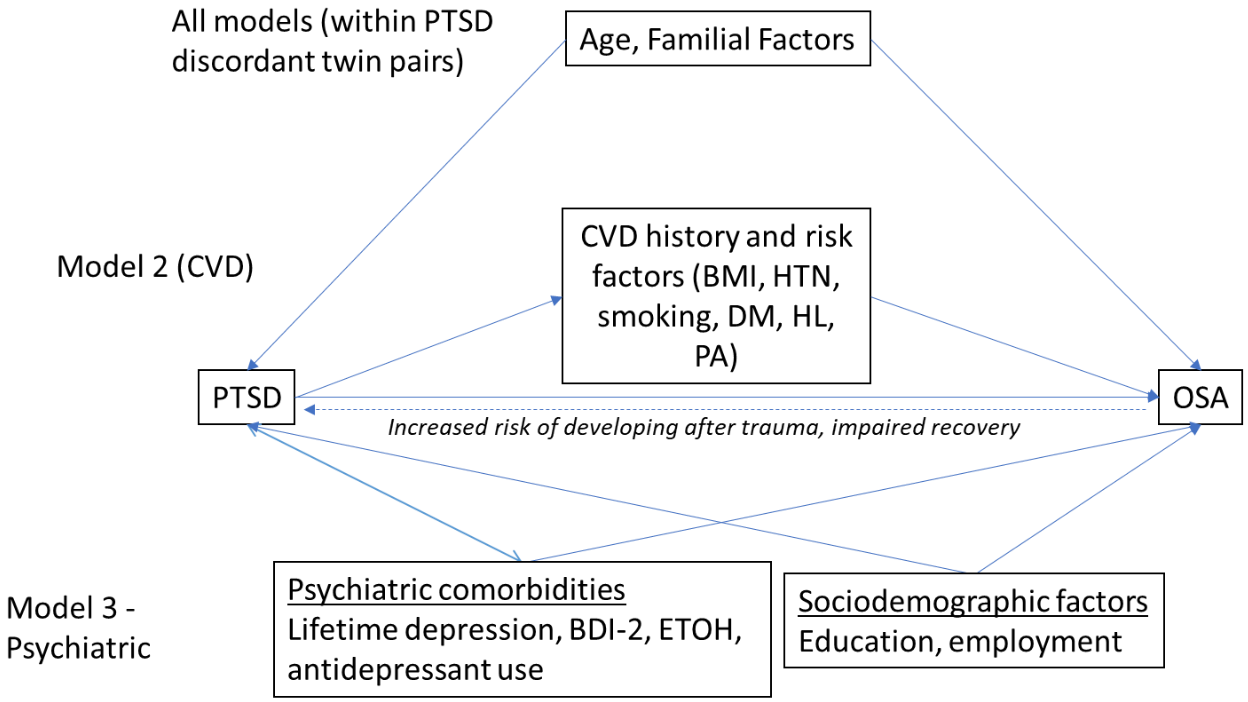

*All models adjust for age and familial factors. In addition, model 2 adjusts for possible CVD risk factors and CVD history, while model 3 adjusts for potential confounders that include demographic and psychiatric factors.

Abbreviations: PTSD=posttraumatic stress disorder; CVD=cardiovascular disease; OSA=obstructive sleep apnea; BDI=Beck Depression Inventory; ETOH=alcohol; BMI=body mass index; HTN=hypertension; DM=diabetes mellitus; HL=hyperlipidemia; PA=Baecke physical activity index

© 2024 Shah AJ et al. *JAMA Network Open.*
