# Supplementary material for: Posttraumatic Stress Disorder and Obstructive Sleep Apnea in Twins
Source: JAMA Netw Open. 2024 Jun 24;7(6):e2416352. doi: 10.1001/jamanetworkopen.2024.16352 (PMC11197451; doi:10.1001/jamanetworkopen.2024.16352)
Supplement: Supplement 2. — Data Sharing Statement [file jamanetwopen-e2416352-s002.pdf]

Data Sharing Statement

Shah. Posttraumatic Stress Disorder and Obstructive Sleep Apnea in Twins. *JAMA Netw Open*. Published June 24, 2024. doi:10.1001/jamanetworkopen.2024.16352

Data
Data available: Yes
Data types: Deidentified participant data
How to access data: We will share the de-identified data upon reasonable request as per institutional policy
When available: beginning date: 12-21-2024

Supporting Documents
Document types: None

Additional Information
Who can access the data: researchers whose proposed use of the data has been approved
Types of analyses: to replicate the findings
Mechanisms of data availability: after approval
Any additional restrictions: as per institutional DUA
